# Disulfiram inhibits TGF-β-induced epithelial-mesenchymal transition and stem-like features in breast cancer via ERK/NF-κB/Snail pathway

**DOI:** 10.18632/oncotarget.5723

**Published:** 2015-10-16

**Authors:** Dan Han, Gang Wu, Chan Chang, Fang Zhu, Yin Xiao, Qiuhui Li, Tao Zhang, Liling Zhang

**Affiliations:** ^1^ Cancer Center, Union Hospital, Tongji Medical College, Huazhong University of Science and Technology, Wuhan, China

**Keywords:** breast cancer, epithelial-mesenchymal transition, cancer stem cells, disulfiram

## Abstract

Disulfiram (DSF), an anti-alcoholism drug, has been reported as an inhibitor of NF-κB. NF-κB is involved in epithelial-mesenchymal transition (EMT) and self-renewal of breast cancer stem cells (CSCs). In this study, we treated MCF-7 and MDA-MB-231 breast cancer cells with TGF-β to induce EMT and cancer stem-like features and studied whether DSF can reverse this process. We found that DSF inhibited TGF-β induced EMT in breast cancer cells in a dose-dependent manner. Also, DSF inhibited EMT-associated stem-like features, migration and invasion of tumor cells as well as tumor growth in xenograft model. The activation of NF-κB was linked with EMT and stem-like cells. We conclude that DSF can suppress NF-κB activity and downregulate ERK/NF-κB/Snail pathway, leading to reverse EMT and stem-like features. Our data suggest that DSF inhibits EMT and stem-like properties in breast cancer cells associated with inhibition of the ERK/NF-κB/Snail pathway.

## INTRODUCTION

Breast cancer is the most common malignant tumor in female, and metastasis is the leading cause of cancer related death [1]. In spite of many advances such as HER-2 or VEGF targeting medicines, median overall survival of patients with advanced breast cancer is still only 2–3 years [2]. Therefore, there is a great need for novel mechanistic understanding of tumor metastasis, which would be critical for developing more effective therapies.

Epithelial–mesenchymal transition (EMT) is a fundamental process for morphogenesis during embryonic development, tissue remodeling and wound healing [3, 4], but more recently it has also been implicated in cancer progression and metastasis [5–7]. Moreover, EMT can be induced by external signals, such as transforming growth factor (TGF)-β, which has been mainly used as an EMT inducer in various experimental studies [8–10]. The most prominent characteristic of EMT is the morphological alteration from epithelial to mesenchymal, which is often accompanied by the downregulated expression of epithelial markers, such as E-cadherin, and upregulated expression of mesenchymal markers, such as vimentin. Emerging evidence suggests that EMT endows cells not only with migratory and invasive properties, but also with stem cell properties [11, 12]. Recent studies have demonstrated that EMT promotes the generation of stem-like cells from differentiated neoplastic cells [13, 14].

Cancer stem cells (CSCs) or cancer stem-like cells [15] have been considered as a crucial role in tumorigenesis, tumor metastasis, chemo- and radiotherapy resistance, and recurrence [16]. Although CSCs constitute a small minority of neoplastic cells within a tumor, they are believed to possess pluripotent and self-renewal capacity, thereby generating a heterogeneous cell population of the originating tumor, seeding at distant sites and driving the formation of macrometastasis. The induction of EMT in immortalized human mammary epithelial cells results in the acquisition of stem-like traits and in the expression of stem cell markers, a CD44^+^/CD24^−^ antigen phenotype [13]. The CD44^+^/CD24^−^ expression pattern has been implicated in both human breast CSCs and normal epithelial stem cells [17, 18]. Aldehyde dehydrogenase (ALDH) is also considered as an important marker for CSCs and refers to the metastasis and recurrence for breast cancer [19, 20]. The cell population bearing both ALDH^+^ and CD44^+^/CD24^−^ CSCs phenotypes has been reported that had high tumorigenic capacity [19]. Targeting both ALDH^+^ and CD44^+^/CD24^−^ phenotypes to eradicate CSCs might be more effective.

It appears that there is a tight link between the EMT and the generation of stem-like cells. Is there any shared signal pathway between these two critical process involving metastasis? Recent studies demonstrated that the transcription factor NF-κB plays an essential role in the induction and maintenance of EMT [21, 22] as well as in the regulation of self-renewal capacity of breast CSCs [23]. Blockade of ERK/NF-κB in peritoneal mesothelial cells has been reported to inhibit the expression of the transcription factor Snail1, a potent inducer of EMT, and revert cells to epithelioid morphology [24]. However, the role of ERK/NF-κB/Snail1 in breast cancer cells has not been elucidated. Investigation of the changes of ERK/NF-κB/Snail pathway in breast cancer during EMT might provide novel strategies for treatment of breast cancer.

As a member of the dithiocarbamate family, disulfiram (DSF) has been safely used for the treatment of alcohol abuse for over sixty years. Increasing evidence suggests that the old drug has a bright new future, exhibiting potent anticancer effects by inducing apoptosis, reducing angiogenesis, suppressing tumor growth, and reversing drug-resistance [25, 26]. Most importantly, as a novel proteasome inhibitor, DSF also inhibits nuclear translocation and DNA binding activity of NF-κB in certain kinds of cancer [25, 27]. It has been demonstrated that the proteasome inhibitor, NPI-0052 renders cells resistant to TGF-α induced EMT by inhibition of NF-κB activation [28]. Thus, we deduced that DSF might have the similar effects on regulation of TGF-β induced EMT as NPI-0052 does. Recent studies reported that DSF can markedly inhibit the proliferation and self-renewal of glioma stem cells by inhibiting NF-κB pathway [29–31]. Moreover, DSF is an irreversible inhibitor of ALDH, and therefore it might also be an inhibitor of ALDH-positive CSCs in breast cancer [32–34]. Nevertheless, whether DSF might overcome both EMT and stem-like characteristics in breast cancer through inhibiting NF-κB and ALDH activity is unknown thus far.

In this study, we demonstrate that DSF can reverse TGF-β induced EMT and stem-like properties of breast cancer cells in *vitro* and in *vivo*. Blockade of ERK/NF-κB/Snail pathway actively suppressed the EMT, migration, invasion, and stem-like features in breast cancer cells. Our results provide supporting evidence to warrant further clinical trial for DSF as a safe and potent anticancer drug.

## RESULTS

### DSF inhibits TGF-β-induced EMT in breast cancer cells

**Figure 1:**
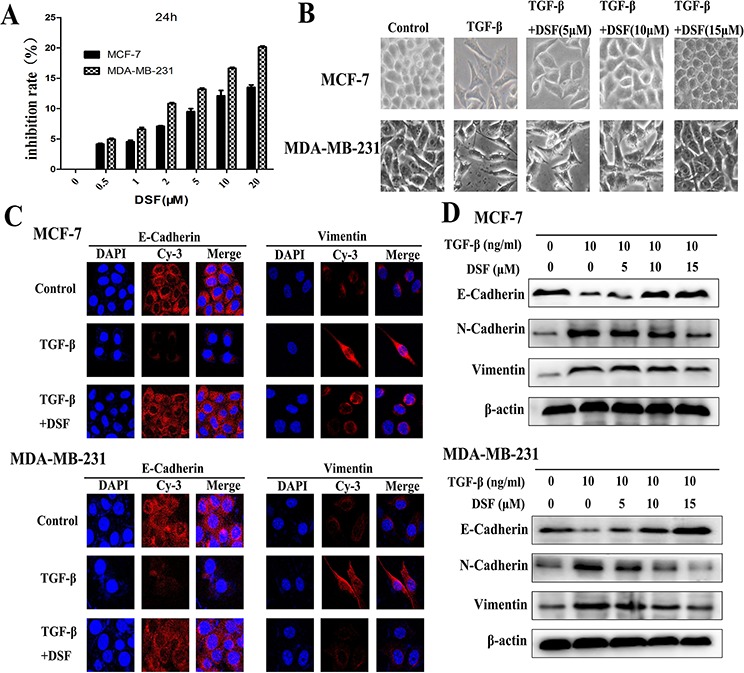
DSF inhibits TGF-β-induced EMT in breast cancer cells **A.** Titration of DSF induced cytotoxicity in MCF-7 and MDA-MB-231 cells. Cells were treated with different concentrations of DSF for 24 h. The inhibition rate of viability of drug-treated cells was determined by MTT assay. Values represent the mean ± SEM of three independent experiments and were calculated based on the control value set at 0 (control: untreated cells). **B.** The morphological change of MCF-7 and MDA-MB-231 cells after exposure to TGF-β (10 ng/ml) alone or combined with DSF (5, 10, 15 μM) under light microscope (× 200 magnification). **C.** Immunofluorescence staining results of epithelial marker E-cadherin and mesenchymal marker vimentin in cells treated with TGF-β (10 ng/ml) alone or combined with DSF (15 μM) (× 400 magnification). **D.** Western blot results of the indicated EMT-related proteins derived from cells treated with TGF-β (10 ng/ml) alone or combined with DSF (5, 10, 15 μM).

To ensure that DSF-blocked TGF-β induced EMT was not due to cell death or inhibition of proliferation, we initially determined subtoxic DSF concentrations for MCF-7 and MDA-MB-231 cells by performing MTT assay (Figure 1A). A range of DSF concentrations (5, 10, 15 μM) were chosen to be used in the following experiments. Cells were pretreated with DSF for 24 h with increasing concentrations (5 μM, 10 μM, 15 μM) prior to stimulation with TGF-β (10 ng/ml) for 24 h. We first observed the morphological change, which is indicative of EMT. As shown in Figure 1B, TGF-β induced MCF-7 and MDA-MB-231 cells undergoing EMT process along with acquiring fibroblast-like, mesenchymal morphology, and DSF treatment inhibited TGF-β induced EMT in a dose dependent manner. After 24 h treatment with increasing concentrations of DSF, these cells kept a more epithelial-like appearance even induced by TGF-β. To further clarify whether abolishment of TGF-β-induced EMT in DSF treated cells resulted from dysregulation of EMT related proteins, we already examined the expression patterns of epithelial marker E-cadherin as well as the expression of the mesenchymal markers, vimentin and N-cadherin. The protein levels were assessed by immunofluorescence staining and western blot assay. As anticipated, DSF significantly suppressed the TGF-β induced upregulation of vimentin and N-cadherin as well as downregulation of E-cadherin in a dose-dependency (Figure 1C and 1D). These findings suggest that DSF can inhibit TGF-β induced EMT by modifying the expression of EMT related proteins.

### DSF targets stem-like cells generated by induction of TGF-β

**Figure 2:**
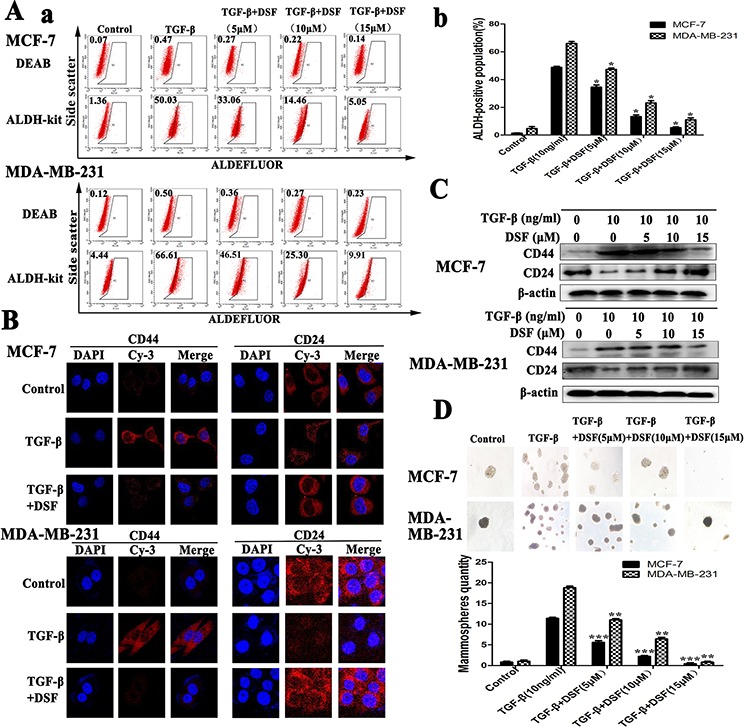
DSF inhibits stem-like properties generated by induction of TGF-β in breast cancer cells **A.** ALDEFLUOR assay. DSF (15 μM) significantly decreased TGF-β (10 ng/ml) induced ALDH^+^ cell population. **B.** Immunofluorescence staining results of CD44 and CD24 expression in cells treated with TGF-β (10 ng/ml) alone or combined with DSF (15 μM) (× 400 magnification). **C.** Western blot results of CD44 and CD24 proteins derived from cells treated with TGF-β (10 ng/ml) alone or combined with DSF (5, 10, 15 μM). **D.** DSF inhibited mammosphere-forming ability in MCF-7 and MDA-MB-231 cells stimulated by TGF-β. Upper panel: The representative images of mammosphere formation assay (× 100 magnification). Values represent the mean ± SEM of three independent experiments.*P < 0.05, **P < 0.01, ***P < 0.001 (in comparison with TGF-β).

There is a tight link between EMT and stem-like cells, where cells undergoing EMT acquire properties of stem cells [14]. In our experiment, induction of EMT in breast cancer cells by exposure of TGF-β resulted in the acquisition of stem-like expression pattern (ALDH^+^, CD24^−^ /CD44^+^) and self-renewal capacity (Figure 2). To determine the targeting effect of DSF on stem-like cells, the expression changes of breast CSCs markers were analyzed. ALDEFLUOR assay indicated that the ALDH^+^ cell population was significantly decreased in DSF treated cells in a dose-dependent fashion. As shown in Figure 2A, TGF-β treatment increased the ALDH^+^ cells from 1.34 ± 0.22% to 48.72 ± 0.67% (*P* < 0.05) in MCF-7 cells and from 4.76 ± 1.13% to 65.88 ± 1.20% (*P* < 0.05) in MDA-MB-231 cells. However, DSF reversed the effects of TGF-β and decreased the ALDH^+^ cells from 48.72 ± 0.67% to 5.31 ± 0.52% (*P* < 0.05) in MCF-7 cells and from 65.88 ± 1.20% to 10.97 ± 1.17% (*P* < 0.05) in MDA-MB-231 cells.

The results of immunofluorescence staining and western blot showed DSF significantly suppressed the TGF-β induced upregulation of CD44 and downregulation of CD24 in a dose-dependency (Figure 2B and 2C). We further investigated the effect of DSF on self-renewal capacity by mammosphere formation assay. When cells were treated with TGF-β, the efficiency of mammospheres forming was significantly increased, whereas the sphere-forming ability was almost completely abolished after 24 h exposure to DSF (Figure 2D). These findings strongly support that DSF is able to inhibit stem-like properties.

### DSF suppresses TGF-β induced cell migration and invasion

**Figure 3:**
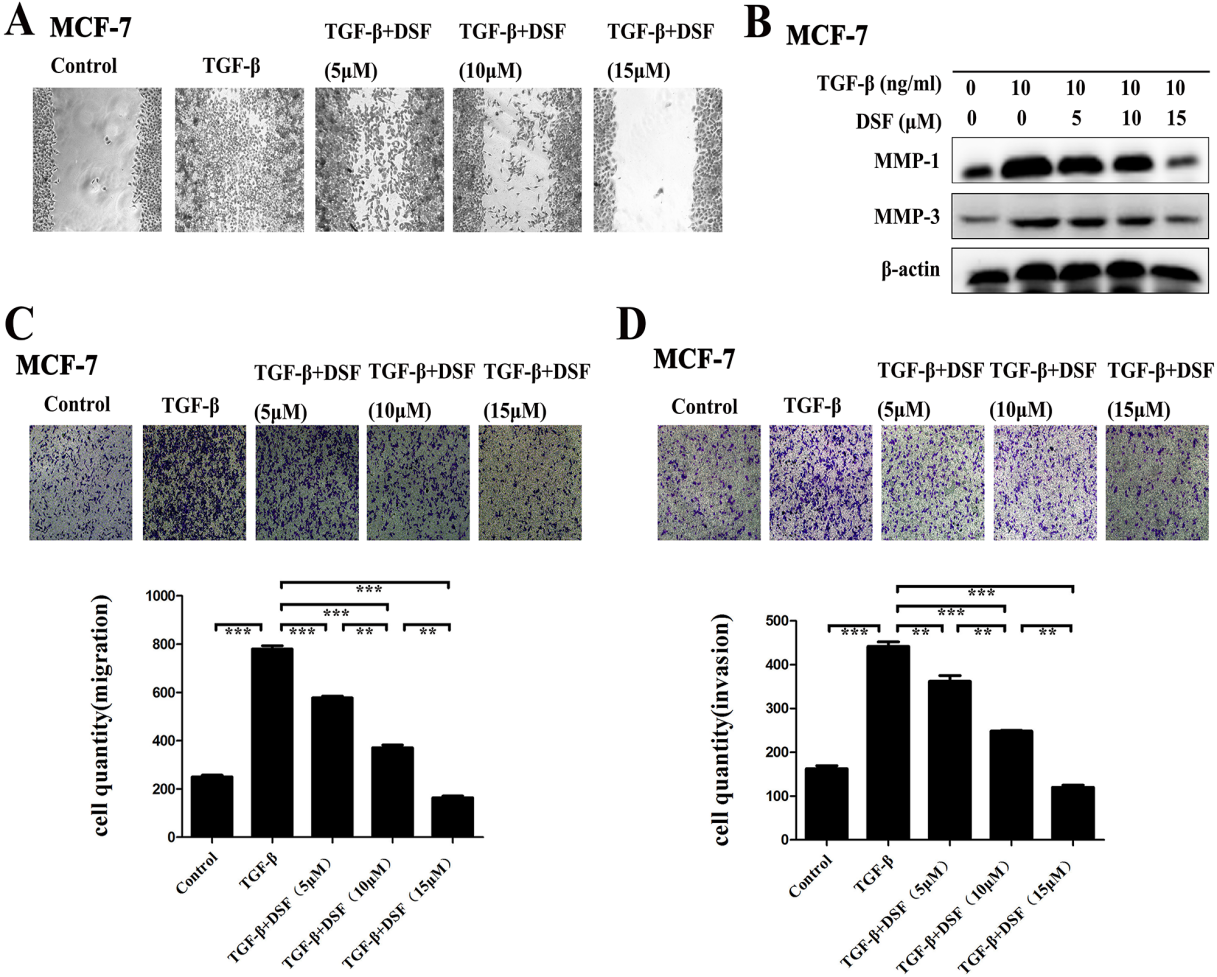
DSF suppresses TGF-β induced cell migration and invasion **A.** Wound- healing assay (× 100 magnification). DSF inhibited the migratory ability of cells stimulated by TGF-β (10 ng/ml). **B.** Western blot results of MMP-1 and MMP-3 proteins derived from cells treated with TGF-β (10 ng/ml) alone or combined with DSF (5, 10, 15 μM). **C** and **D.** Transwell-based migration and invasion assay. DSF inhibited the migratory (C) and invasive (D) properties of cells stimulated by TGF-β (10 ng/ml). Values represent the mean ± SEM of three independent experiments. Upper panels: representative pictures of migratory or invade cells (blue staining) under different treatment conditions (× 100 magnification). **P < 0.01, ***P < 0.001.

The functional significance of DSF inhibiting the expression profiles of the above EMT-related gene products was expected to be reflected in the cell migration and invasion. To evaluate the alteration of tumor cell migratory and invasive properties, wound-healing and transwell-based assays were performed. As anticipated, DSF significantly suppressed both the tumor cell migration (Figure 3A and 3C) and invasion (Figure 3D). TGF-β promoted MCF-7 cells migration and invasion, whereas this tendency was blocked by DSF in a dose-dependent manner. We further detected the invasion-related proteins MMPs (MMP-1 and MMP-3). The data indicated that DSF significantly suppressed the TGF-β induced upregulation of MMP-1 and MMP-3 (Figure 3B). These results suggest that the induction of TGF-β could encourage migration and invasion, and this process could be dramatically blocked by DSF.

### DSF inhibits ERK/NF-κB/Snail pathway

**Figure 4:**
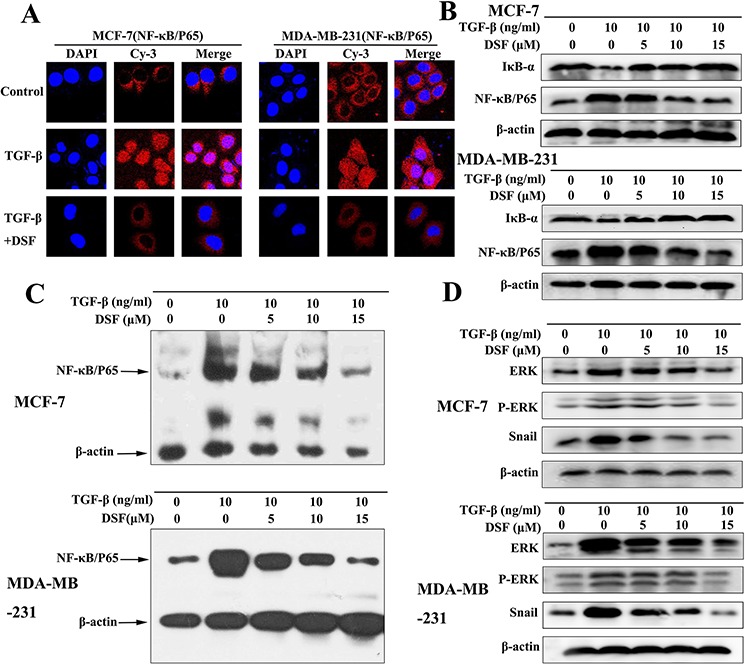
DSF inhibits ERK/NF-κB/Snail pathway **A.** Immunofluorescence staining shows that DSF (15 μM) inhibited TGF-β (10 ng/ml) induced p65 nuclear translocation (× 400 magnification). **B.** Western blot results of NF-κB p65 and IκBα proteins derived from cells treated with TGF-β (10 ng/ml) alone or combined with DSF (5, 10, 15 μM). **C.** EMSA results of nuclear extract derived from cells at different treatment conditions. **D.** The effect of DSF on the upstream signaling (ERK, p-ERK) and downstream signaling (Snail) of NF-κB was determined by western blot.

Increasing evidence suggests that transcription factor NF-κB plays a critical role in the induction and maintenance of EMT, as well as in the expansion of breast CSCs [20–22]. Therefore, we examined the inhibitory effect of DSF on NF-κB by assessing its nuclear translocation and DNA binding activity. Shown in Figure 4A, NF-κB p65 nuclear translocation was induced by TGF-β, while TGF-β induced p65 nuclear translocation was blocked by DSF. Western blot results showed that DSF suppressed the TGF-β induced upregulation of NF-κB p65 protein and prevented the TGF-β triggered IκBα degradation (Figure 4B). The NF-κB p65 nuclear translocation and IκBα degradation critically influence NF-κB DNA binding activity. EMSA results showed DSF inhibited TGF-β mediated NF-κB DNA binding affinity (Figure 4C).

We analyzed upstream signaling that might attribute to NF-κB activation in our experimental setting. The activation of the ERK contributes to the regulation of NF-κB activity during TGF-β induced EMT [35]. As expected, TGF-β treatment increased the expression of ERK and phosphorylation of ERK. However, these effects were blocked by DSF (Figure 4D). We also analyzed downstream signaling of NF-κB that might account for TGF-β induced EMT and stem-like cells. Snail has been reported to be upregulated partly by NF-κB and TGF-β [36, 37], and it is a transcription factor involving EMT and CSC regulations [38]. Our data indicated that the expression of Snail was induced by TGF-β, but suppressed by DSF in a dose-dependent manner (Figure 4D). These findings suggest that DSF inhibits the ERK/NF-κB/Snail pathway, resulting in inhibition of TGF-β-mediated EMT, self-renewal, migration and invasion.

### Direct role of ERK/NF-κB/Snail pathway repression on the reverse of TGF-β induced EMT, stem-like properties, migration and invasion

**Figure 5:**
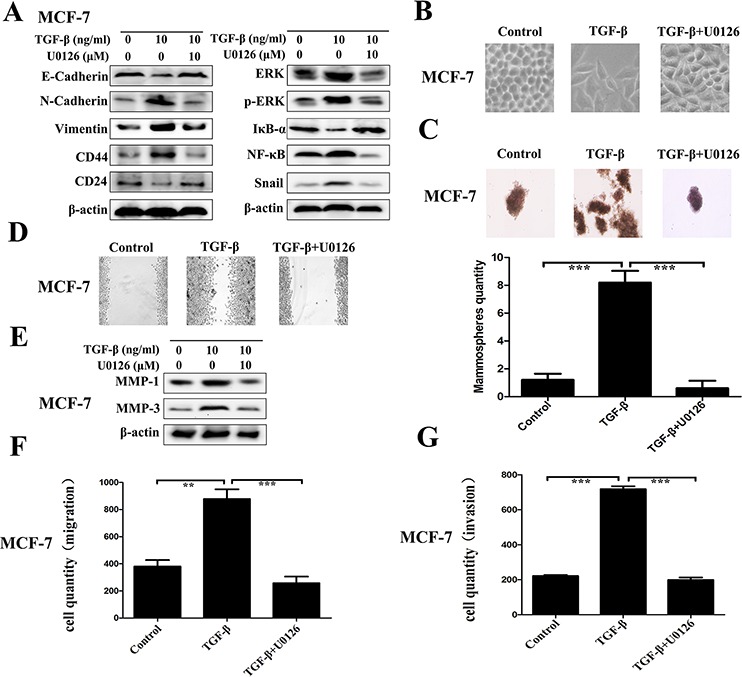
Repression of ERK/NF-κB/Snail pathway results in inhibition of TGF-β induced EMT, stem-like properties, migration and invasion The direct role of ERK/NF-κB/Snail pathway was determined by using U0126, a chemical inhibitor of MEK1/2 - the upstream activator of ERK. **A.** Western blot results of EMT-related, CSCs-related, and ERK/NF-κB/Snail pathway-related proteins derived from cells treated with TGF-β (10 ng/ml) alone or combined with U0126 (10 μM). **B.** The morphological change of MCF-7 cells after exposure to TGF-β (10 ng/ml) alone or combined with U0126 (10 μM) (× 200 magnification). **C.** The morphology of mammosphere formation assay (× 100 magnification). **D.** Wound-healing assay (× 100 magnification). **E.** Western blot results of MMP-1 and MMP-3 proteins derived from cells treated with TGF-β (10 ng/ml) alone or combined with U0126 (10 μM). **F.** Transwell-based migration assay. **G.** Transwell-based invasion assay. Values represent the mean ± SEM of three independent experiments. **P < 0.01, ***P < 0.001.

The direct role of ERK/NF-κB/Snail pathway repression on the regulation of TGF-β induced EMT, stem-like properties, migration and invasion was determined by using U0126, a chemical inhibitor of MEK1/2 - the upstream activator of ERK. As Figure 5A shown, it is easy to observe that U0126 blocks the ERK/NF-κB/Snail pathway surely with related proteins ERK, p-ERK, NF-κB and Snail downregulation, accompanied with IκB-α accumulation. Concomitant with the effect of DSF on the expression profiles of EMT and CSCs, U0126 treatment markedly inhibited the TGF-β induced EMT morphologically (Figure 5B) and biochemically, along with increased expression of E-cadherin and reduced levels of N-cadherin and vimentin (Figure 5A). U0126 also inhibited the expression of breast CSCs markers with upregulation of CD24 and downregulation of CD44, along with suppressing self-renewal capability of TGF-β treated cell (Figure 5A and 5C). Moreover, U0126 significantly suppressed the TGF-β induced migration and invasion with downregulation of MMP-1 and MMP-3 (Figure 5D–5G). These results corroborated the above findings in DSF treated cells and confirmed the role of ERK/NF-κB/Snail pathway in the inhibitory effect of DSF on the EMT and CSCs phenotypes.

### DSF inhibits tumor growth and metastasis *in vivo*

**Figure 6:**
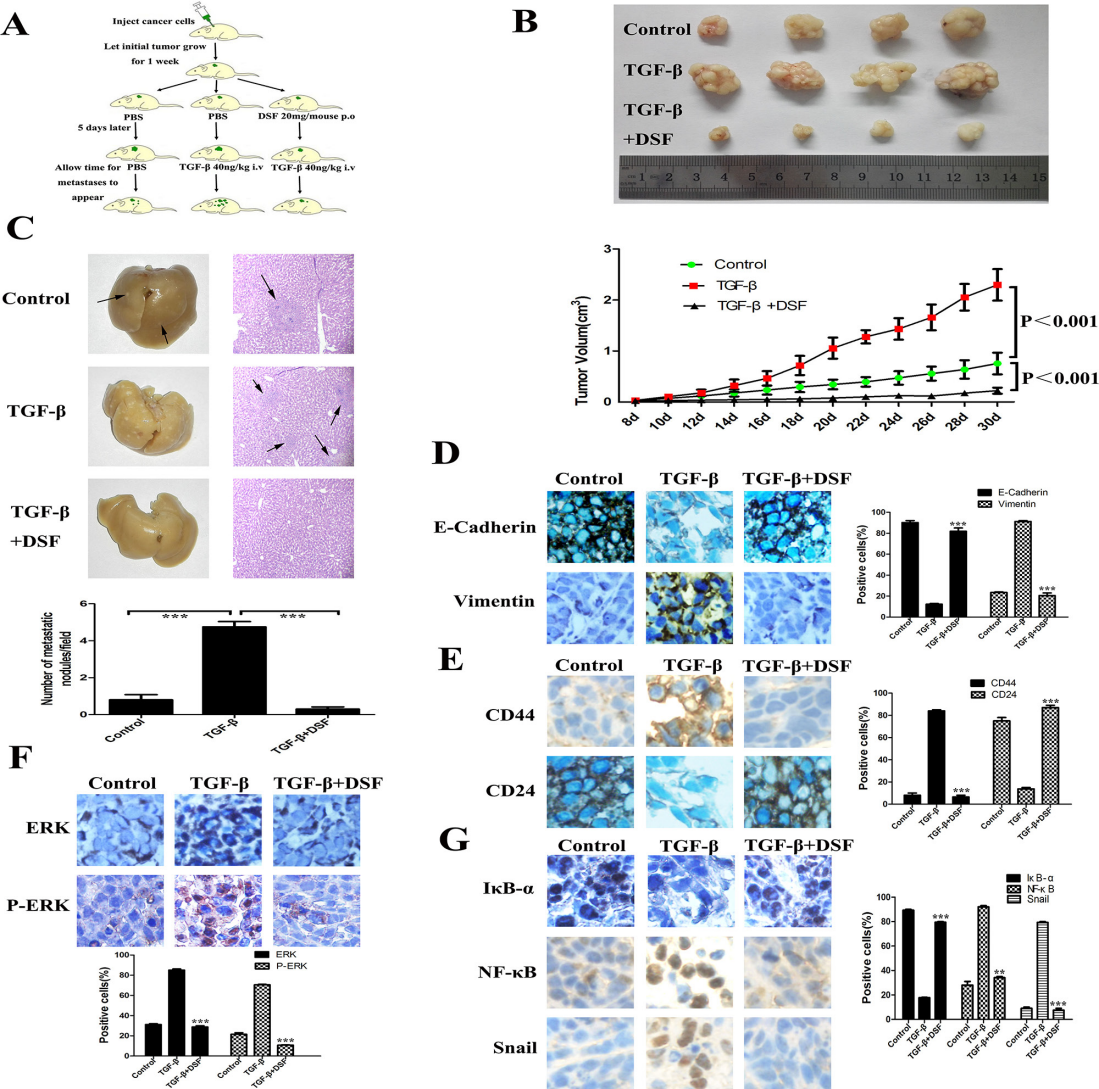
DSF inhibits tumor growth and metastasis *in vivo* **A.** Flowchart of animal experiments. MCF-7 cells (1 × 10^7^) were subcutaneously injected in the right flank of mice. After one week of injection, the tumor bearing mice were randomly subdivided into 3 groups (5 mice/group): control; TGF-β 40 ng/kg i.v; DSF 20 mg/mouse p.o + TGF-β 40 ng/kg i.v. **B.** Upper panel: Representative photographs of the tumors produced by MCF-7 cells. Lower panel: Growth curves of tumor size. Values represent the mean ± SEM. **C.** Metastatic tumor nodules in the liver were examined by H & E staining of serial sections. Tumor nodules are marked with arrows (× 100 magnification). Lower panel: The numbers of cancerous metastatic nodules in these liver sections were counted and the average number per field of view is presented. ***P < 0.001. **D.** The effect of different treatment on E-cadherin and vimentin expression (× 200 magnification). **E.** The effect of different treatment on CD44 and CD24 expression (× 200 magnification). **F** and **G.** The effect of different treatment on ERK, p-ERK, IκBα, NF-κB and Snail expression (× 200 magnification). **P < 0.01, ***P < 0.001 (in comparison with TGF-β).

To investigate the inhibitory effects of DSF on tumor growth and metastasis *in vivo*, subcutaneous xenograft nude mouse model was used. The schedule of animal experiments was shown in a flowchart (Figure 6A). We found that TGF-β significantly promoted tumor growth as compared to the control group, but DSF strongly inhibited this tendency (Figure 6B). The data suggest that exogenous TGF-β facilitates tumor growth as it happens *in vitro*, but DSF efficiently inhibits tumor growth, which completely eliminates the effect of TGF-β. Importantly, we found significantly fewer metastatic nodules in liver tissues of mice treated with DSF than in mice treated with PBS or TGF-β. Metastases in the liver of mice carrying tumors were confirmed by H& E staining (Figure 6C, upper). The average tumor metastatic nodules per field were 4.75 in TGF-β group, whereas nearly no metastatic nodules (*N* = 0.3) was detected in DSF group (Figure 6C, lower) (*P* < 0.001).

We next performed immunohistochemical detection of EMT and CSCs markers in tumor tissues. The changes of epithelial and mesenchymal makers and loosely organization structures indicated that exogenous TGF-β could indeed arouse EMT *in vivo*, along with the activation of ERK/NF-κB/Snail pathway. As it did *in vitro*, DSF treatment reversed TGF-β induced EMT related proteins expression and downregulated ERK/NF-κB/Snail pathway (Figure 6D and 6G). DSF also suppressed TGF-β induced CD44 upregulation and CD24 downregulation (Figure 6E), suggesting that DSF may inhibit stem-like features *in vivo* but further studies are needed. Taken together, these data suggest that DSF suppresses tumor growth and metastasis *in vivo* via ERK/NF-κB/Snail pathway.

## DISCUSSION

Metastasis is the leading cause responsible for treatment failure in breast cancer. Both the EMT and CSCs are considered as a crucial role contributing to tumor recurrence and metastasis [39]. Growing evidence suggests that EMT endows cells not only with migratory and invasive properties, but also with stem cell properties [11–14, 40]. Agents that inhibit the EMT program may serve as dual inhibitors by not only inhibiting tumor cell invasion and metastasis but also inhibiting the generation of stem-like cells via EMT. In the present study, we found that DSF, a drug used to treat alcoholism, is such an agent that may serve as the dual inhibitors for EMT and CSCs. Our results demonstrated that DSF can effectively reverse TGF-β induced EMT and stem-like properties of breast cancer cells by targeting NF-κB signaling pathway *in vitro* and *in vivo*.

Although the anticancer effects of DSF have been known for many years [41], very little is known about the activity of this drug on EMT and stem-like cells in breast cancer. First, we examined the potential EMT-inhibitory activity of DSF by checking the cellular morphological changes and alterations of EMT-related markers expression. We found that DSF can reverse the TGF-β induced EMT program in a dose-dependent manner *in vitro* and in mice model. Second, we examined the potential inhibitory effect of DSF on stem-like properties in breast cancer cells. Our results showed that DSF can inhibit the self-renewal capability of breast cancer cells, and reverse the expression of breast CSC markers, such as ALDH, CD44 and CD24. Third, we found that DSF effectively inhibited tumor cell migration and invasion through modulation of MMP-1 and MMP-3 expression. DSF also significantly inhibited tumor growth and metastasis in mice model. Of notable importance, our data indicate that DSF alone was highly effective to against EMT and cells with stem-like properties in breast cancer.

Previous studies demonstrate that the anticancer activity of DSF was in a metal (especially copper)-dependent manner. Since DSF is a member of the dithiocarbamate family, as a chelator, DSF strongly chelates copper, zinc and some other bivalent metals to form DSF/bivalent metal-complex [42, 43]. In combination with copper, DSF forms DSF/copper complex which is highly cytotoxic to breast cancer, glioblastoma, melanoma and colon cancer cells [29, 32, 43, 44]. In addition, DSF/copper complex was reported to be able to induce apoptosis, reduce angiogenesis, suppress tumor growth, and reverse drug-resistance [26]. However, most recent studies revealed that both DSF/copper complex and DSF alone appear to effectively target glioblastoma and breast cancer cells with cancer-stem-like properties [29–34]. DSF alone, even at a concentration as low as 100 nM, suppressed self-renewal of glioblastoma and was highly effective in cells that are refractory to temozolomide [30]. Studies in breast cancer reported that DSF may target breast cancer cells with CSC-like properties by modulating NF-κB pathway [32, 34]. These findings are in line with our results. Here, we demonstrated the inhibitory activity of DSF on breast cancer stem-like cells in the absence of metal supplementation. Furthermore, to our best knowledge, this is the first time to report the efficacy of DSF on inhibition of EMT in breast cancer.

The molecular anticancer mechanisms of DSF remain unclear. Our data showed that DSF inhibited NF-κB activity by preventing degradation of its inhibitor IκBα and inhibiting its nuclear translocation and DNA binding activity. Our results are in line with previous studies. DSF has been reported as a novel proteasome inhibitor, and its inhibitory effects on NF-κB pathway have been investigated in colorectal cancer, glioblastoma, and breast cancer [25, 29, 43]. In recent studies, DSF has been shown potent inhibition of breast CSCs by simultaneous induction of ROS and inhibition of NF-κB [32, 34]. NF-κB plays a critical role in the induction and maintenance of EMT, as well as in the regulation of self-renewal capacity of breast CSCs. The activation of the ERK contributes to the regulation of NF-κB activity during TGF-β induced EMT [24, 35]. Blockade of ERK/NF-κB/Snail can reverse TGF-β induced EMT in peritoneal mesothelial cells [24]. Our results demonstrated the inhibitory effect of DSF on ERK/NF-κB/Snail pathway, and direct role of inhibition of ERK/NF-κB/Snail on the regulation of EMT and CSCs in breast cancer was tested by U0126, a chemical inhibitor of MEK1/2 - the upstream activator of ERK. Concordant with the effects of DSF on the expression profile of EMT and CSCs, U0126 treatment leaded to the reversion of EMT and stem-like properties, and the suppression of invasion and migration. These findings strongly suggested that DSF inhibited EMT and cancer stem-like cells by targeting ERK/NF-κB/Snail pathway.

DSF is widely reported as an inhibitor of ALDH, a surrogate marker of CSCs [19]. Our results demonstrated that DSF treatment suppressed the expression of breast CSCs markers (ALDH^+^, CD44^+^/CD24^−^), and abolished the sphere-forming ability in breast cancer cells at a concentration of 10–15 μM. However, several studies reported that DSF alone did not show any inhibitory effect on ALDH activity [29, 32]. The discrepancy between others and this study may be due to the different DSF concentrations. Much lower concentrations (500 nM, 1 μM) were used in these studies for evaluation of DSF against CSCs. Although data obtained here suggest that DSF may inhibit ALDH positive cell populations, further studies are needed to evaluate the contribution of ALDH activity to breast CSCs death. It also should be noted that aldehyde dehydrogenases are a superfamily with 19 isoenzymes. The ALDH1A1 and ALDH1A3 isoforms have been identified as major markers of breast CSCs [19, 45]. However, it was recently demonstrated that DSF/copper has potent inhibitory action on ALDH enzyme activity, but has no effect on the mRNA and protein expression of ALDH isoeznymes (1A1, 1A3, 2 and 3A1) [34]. Triscott and colleagues [30] also reported that DSF inhibits ALDH activity, but its effect on glioblastoma stem-like cells may be not through ALDH inhibition.

DSF has been safely used to control alcoholism for over sixty years, and its anticancer effects are being investigated for other malignancies in clinical trials. A phase II trial is evaluating the use of DSF in newly diagnosed glioblastoma multiform (http://ClinicalTrials.gov Identifier: NCT01777919). Another phase II trial investigated the effect of DSF in combination with standard chemotherapy in non-small cell lung cancer (http://ClinicalTrials.gov Identifier: NCT00312819). In the present study we indicated that DSF can potentially reverse EMT and stem-like properties of breast cancer cells via the ERK/NF-κB/Snail pathway *in vitro* and *in vivo*. Clinical studies of DSF as a novel anticancer agent in breast cancer seem warranted.

## MATERIALS AND METHODS

### Cell lines and cell culture

Human breast cancer cell lines MCF-7 and MDA-MB-231 were obtained from ATCC (Manassas, VA, USA). Cells were cultured in Dulbecco's modified Eagle medium (DMEM, Hyclone) supplemented with 10% heat-inactivated fetal bovine serum (FBS, Hyclone), in a 37°C humidified environment containing 5% CO_2_.

Cells were cultured to 60% confluence, and subsequently exposed to DSF at indicated concentrations for 24 h followed by TGF-β (10 ng/ml) treatment for another 24 h.

### Reagents and antibodies

DSF was purchased from Sigma (USA) and dissolved in DMSO. TGF-β was purchased from Peprotech (USA) and dissolved in 10 mM citric acid, PH3.0 to a concentration of 0.1–1.0 mg/ml. U0126 was purchased from Selleck Chemicals (USA) and dissolved in 10% DMSO, 30% Cremophor EL and 60% PBS to a concentration of 10 mM.

The following primary antibodies were used: anti-E-cadherin (WB: 1:1000, IF:1:200, IHC:1:400 dilution, CST), anti-vimentin (WB: 1:1000, IF:1:100, IHC:1:100 dilution, CST), anti-N-cadherin (WB: 1:1000 dilution, CST), anti-Snail (WB: 5 ng/ml, IF:10 μg/ml, IHC: 10 μg/ml, R&D Systems), anti-NF-κB/p65 (WB: 1:1000, IF:1:50, IHC:1:800 dilution, CST), anti-ERK (WB: 1:500, IF:1:50, IHC:1:50, dilution, Boster, Wuhan, China), anti-p-ERK (WB: 1:1000, IF:1:200, IHC:1:400 dilution, CST), anti-IκB-α (WB:1:500, IF: 1:50, IHC:1:50 dilution, Anbo, USA), anti-CD24 (WB: 1:200, IF:1:50 dilution, Santa Cruz), anti-CD24 (IHC:1:50 dilution, Abcam), anti-CD44 (WB:1:1000, IF:1:200, IHC:1:50 dilution, CST), anti-MMP-1 (WB:1:200 dilution, Santa Cruz), anti-MMP-3 (WB:1:200 dilution, Santa Cruz).

### Cytotoxicity assay

For *in vitro* cytotoxicity assay, 3-(4, 5-dimethylthiazol-2-yl)-2, 5- diphenyltetrazolium bromide (MTT) was used. Cells (10^4^/per well) were cultured overnight in 96-well plates, then treated with DSF in indicated concentrations for 24 h, and subjected to a standard MTT assay.

### Immunofluorescence staining

Cells were cultivated in 24-well plates and treated by drugs. Then the cells were washed with PBS twice and fixed with 4% paraformaldehyde for 30 minutes. After permeabilization with 0.1% Triton X-100, cells were blocked with 5% BSA for 1 h, subsequently incubated with primary antibodies overnight. After washed with PBS, the cells were incubated with Cy3-conjugated goat anti-rabbit or anti-mouse IgG antibody (1:150 dilution, Boster, Wuhan, China) in the dark for 1 h at 37°C, counterstained with DAPI. Images were collected using an Olympus Fluoview FV1000 laser-scanning confocal microscope.

### Western blot

The cells were trypsinized and cultured in 6-well plates and treated by drugs. The cells were washed with 4°C PBS twice and lysed in RIPA Buffer. Then the lysate was centrifuged for 15 minutes and the supernatants retained. The protein extracts were separated on 12% SDS-PAGE and transferred to PVDF membranes. Membranes were blocked with 5% nonfat milk in 0.1% TBST for 1 h and then incubated with primary antibodies as previously described over night at 4°C, followed by incubation with secondary antibodies (1:5000 dilution, Boster, Wuhan, China). The signals were detected by SuperSignal West Pico Chemiluminescence Substrate (Pierce, USA). Equal protein sample loading was monitored by probing the same membrane with antibodies against β-actin.

### Wound-healing assay

Cells were seeded in a 6-well plate to form a confluent monolayer in complete medium. The monolayer was pretreated with the indicated concentrations of DSF for 24 h before being scratched by a plastic tip. Wounded monolayers were washed by PBS to remove cell debris, followed by exposure to TGF-β (10 ng/ml) for another 24 h. Wound closure was monitored under microscopy at × 100 magnification.

### *In vitro* migration and invasion assays

Transwell (Corning Costar, Cambridge, MA, USA) upper chamber were coated with matrigel (BD, USA) for invasion assay or without matrigel for migration assay. The cells were incubated with the indicated concentrations of DSF for 24 h and seeded in the upper well (1 × 10^6^/well) with DMEM medium containing 1% BSA and TGF-β (10 ng/ml), while medium containing 10% FBS in the lower chamber. After incubating for 24 h at 37°C, cells in the upper chamber were carefully removed with a cotton swab, and the invaded cells that had traversed to reserve face of the membrane were fixed with 4% paraformaldehyde and stained with gentian violet.

### Mammosphere culture

MCF-7 and MDA-MB-231 cells were cultured in 6-well plates and exposed to drugs as mentioned above. The cells were collected and furthered cultured in ultra-low adherence 24-well plates (Corning, MA, USA) with 1 ml stem cell culture medium (serum-free DMEM-F12 supplemented with B27 (Invitrogen), 20 ng/ml epidermal growth factor (Peprotech), 10 ng/ml basic fibroblasts growth factor (Peprotech), 10 μg/ml insulin (Sigma)) at a density of 10,000 cells/well. After 7–10 days culture, the mammospheres were photographed.

### ALDEFLUOR assay

The ALDH-positive population in drug treated cells was detected by ALDEFLUOR kit (StemCell Tech., USA) following the manufacturer. The cells (1 × 10^6^/ml) were assayed on a flow cytometer after staining in ALDH substrate containing assay buffer for 30 min at 37°C. The negative control was treated with diethylaminobenzaldehyde (DEAB), a specific ALDH inhibitor.

### Electrophoretic mobility-shift assay (EMSA)

NF-κB DNA-binding activity was measured by EMSA by using a LightShift chemiluminescent EMSA kit (Pierce, USA) as previously described [31]. Briefly, nuclear extract (5 μg) was incubated with biotin-labeled oligonucleotide in binding buffer for 20 min at room temperature. The specificity of the NF-κB DNA-binding was determined by in competition reactions in which a 200-fold molar excess of unlabeled probe was added to the binding reaction. Products of binding reactions were then subjected to gel electrophoresis on a 5.5% polyacrylamide gel and transferred to a PVDF membrane. The signal was detected by chemiluminescent photography.

### Animal experiments

Four- to six-week-old female BALB/c nu/nu mice (Beijing HFK Bioscience, China) were housed under pathogen-free conditions according to the animal care guidelines of Huazhong University of Science and Technology (HUST). The animal experiments were reviewed and approved by the Ethical Committee of HUST. MCF-7 cells (1 × 10^7^) suspending in medium were subcutaneously injected in the right flank of mice. MCF-7 tumor growth was sustained by estrone administered at 1 mg/l in the animals' water supply. The tumor bearing mice were randomly subdivided into 3 groups (5 mice/group): control; TGF-β 40 ng/kg i.v; DSF 20 mg/mouse p.o + TGF-β 40 ng/kg i.v. After one week of injection, the mice were administrated orally with DSF for three consecutive days, while the control and TGF-β groups received PBS. Five days later, the groups (TGF-β group, TGF-β + DSF group) were administrated with TGF-β and the control group was still given PBS. Tumor dimensions were measured with vernier caliper and tumor volumes were estimated by the formula: length × width^2^ × 0.5. The mice were sacrificed after 30 days, and the tumors and the organs with visible metastatic tumors were removed, photographed and subjected to further analysis.

### Immunohistochemistry

Immunohistochemistry was performed on formalin-fixed paraffin-embedded tumor tissue sections. The sections were deparaffinized, rehydrated and stained with primary antibodies over night at 4°C. These antibodies were detected with biotinylated secondary antibody, followed by incubation with horseradish peroxidase-conjugated streptavidin-biotin complex. Finally, the sections were developed in diaminobenzidine and visualized under a light microscope

### Statistical analysis

Data were expressed as mean ± SEM. Each value is the mean of at least three separate experiments in each group. Student's *t*-test was conducted using GraphPad Prism software. *P* < 0.05 was considered significant.
